# Innovative and conventional “conservative” technologies for the treatment of uterine fibroids in Italy: a multidimensional assessment

**DOI:** 10.1186/s13561-022-00367-x

**Published:** 2022-03-18

**Authors:** L. Ferrario, E. Garagiola, C. Gerardi, G. Bellavia, S. Colombo, C. Ticca, C. Rossetti, M. Ciboldi, M. Meroni, A. Vanzulli, A. Rampoldi, T. Bignardi, F. Arrigoni, E. Porazzi, E. Foglia

**Affiliations:** 1 Centre for Health Economics, Social and Health Care Management, LIUC- Università Cattaneo, Corso Matteotti, 22, 21053 Catellanza, VA Italy; 2 IRCCS- Istituto di Ricerche Farmacologiche “Mario Negri”, Milan, Italy; 3 ASST Grande Ospedale Metropolitano Niguarda, Milan, Italy; 4 University of L’Aquila, L’Aquila, Italy

**Keywords:** Economic evaluation, Technology assessment, Magnetic resonance imaging/methods, Ultrasound therapy/methods, Decision support techniques

## Abstract

**Background:**

To evaluate the potential benefits of the Magnetic Resonance-guided high intensity Focused Ultrasound (MRgFUS) introduction in the clinical practice, for the treatment of uterine fibroids, in comparison with the standard “conservative” procedures, devoted to women who wish to preserve their uterus or enhance fertility: myomectomy and uterine artery embolization (UAE).

**Methods:**

A Health Technology Assessment was conducted, assuming the payer’s perspective (Italian National Healthcare Service). The nine EUnetHTA Core Model dimensions were deeply investigated, by means of i) a literature review; ii) the implementation of health economics tools (useful for uterine fibroids patients’ clinical pathway economic evaluation, and budget impact analysis), to define MRgFUS economic and organizational sustainability, and iii) administration of specific questionnaires filled by uterine fibroids’ experts, to gather their perceptions on the three possible conservative approaches (MRgFUS, UAE and myomectomy).

**Results:**

Literature revealed that MRgFUS would generate several benefits, from a safety and an efficacy profile, with significant improvement in symptoms relief. Advantages emerged concerning the patients’ perspective, thus leading to a decrease both in the length of hospital stay (*p*-value< 0.001), and in patients’ productivity loss (*p*-value = 0.024). From an economic point of view, the Italian NHS would present an economic saving of − 6.42%. A positive organizational and equity impact emerged regarding the capability to treat a larger number of women, thus performing, on average, 131.852 additional DRGs.

**Conclusions:**

Results suggest that MRgFUS could be considered an advantageous technological alternative to adopt within the target population affected by uterine fibroids, demonstrating its economic and organisational feasibility and sustainability, with consequent social benefits.

**Supplementary Information:**

The online version contains supplementary material available at 10.1186/s13561-022-00367-x.

## Introduction

Magnetic Resonance-guided high intensity Focused Ultrasound (MRgFUS) is an emerging not-invasive procedure that applies the energy deriving from ultrasound to targeted soft tissues, within the human body, to heat and destroy diseased or damaged tissues, through ablation.

MRgFUS has been approved for use and is currently employed to treat uterine fibroids (UFs), worldwide. Since it was first approved in the U.S. by the Food and Drug Administration in 2004, MRgFUS has represented a new approach for UF treatment [1–3], broadening the range of treatment options for patients, thus being a fertility-preserving technique [4].

UFs are common in women of reproductive age, affecting on average 13.80% of the female population in Italy [5]. The “standard” treatment procedures used to date, have been invasive surgical approaches, such as laparoscopy, open myomectomy, or hysterectomy, that often require a long recovery time and create the risk of complications, significantly reducing the patient’s quality of life, and increasing the National Healthcare Service (NHS) costs.

Clinical studies demonstrate that MRgFUS is a safe and effective treatment for symptomatic uterine fibroids [6–8], with a significant improvement in clinical symptoms in 70–80% of women affected [8–10]. The efficacy profile of MRgFUS was amply rewarded in literature [1–3], in terms of both fibroid volume reduction and symptoms relief, even considering short term follow-up. However, little is known about the economic feasibility of its real-life adoption into the clinical practice, with evidence-based information, only concerning its cost-effectiveness estimation [11–13].

At present, there still exists a debate due to uncertainty about the optimum management of UFs. In particular, in Italy, no consensus exists with regard to the introduction of MRgFUS in the clinical practice, due to a paucity of real-world data, as well as the hospital consequences related to its acquisition, concerning not only economic aspects, efficacy or safety, but also other implications that have acquired importance over the time, such as organizational, equity and social domains, since not only cost-effectiveness assessment [14] is important to define value and benefits of a healthcare technology. In this view, it could be interesting to understand if MRgFUS would bring advantages able to increase patients’ health or to reduce hospitalization time.

Therefore, an in-depth study is needed, particularly in the Italian and in European setting, to produce real-world evidence concerning this peculiar topic, and evaluating all the comparative alternative technologies, useful to solve the UF clinical problem, focusing the attention on the not-invasive approaches, devoted to women who wish to preserve their uterus or enhance fertility.

Moving on from these premises, the present study aims at evaluating, with a multidimensional approach, the benefits concerning the introduction of MRgFUS in Italy, in comparison with other conservative and widely diffused procedures, such as myomectomy (standard surgery) or Uterine Artery Embolization (UAE), whose specific managements are very different in terms of activities, outcomes and costs.

The coverage of this knowledge gap could be useful to support the evidence-based decision-making and reimbursement process, both at hospital and at National level, not only in the Italian context, but also in other European National Healthcare Services.

## Methods

The present study was structured as a Health Technology Assessment (HTA) analysis and it was conducted assuming the Italian NHS point of view, focusing on the comparation between the three different conservative technologies available for the treatment of uterine fibroids, devoted to child-bearing age women, who wish to preserve their uterus (myomectomy, UAE and MRgFUS). Data refer to the year 2018.

Due to the multidimensional and multidisciplinary nature of HTA, several aspects were considered, as stated within the EUnetHTA Core Model [15]: i) general relevance; ii) safety; iii) efficacy and effectiveness; iv) economic and financial impact; v) equity; vi) legal aspects; vii) social and ethical impact; and viii) organizational impact.

For the assessment of the above dimensions, data were gathered, according to a mixed-method approach, a relevant methodology used for healthcare services research, to give a more comprehensive understanding of complex interventions [16, 17]. The following data sources were considered:1) a literature review, to retrieve evidence-based information with regard to the safety and the efficacy profiles related to the alternative technologies under assessment, as well as quality of life measures; 2) quantitative approaches, for economic evaluation and budget impact analysis, as well as for the definition of the organizational impacts, based on real-word data; and iii) administration of specific questionnaires to uterine fibroids’ experts, by means of structured interviews, gathering their perceptions with regard to organizational, social, equity and legal domains, according to the EUnetHTA Core Model requirements [15]. It should be noted here that for the definition of the economic and organizational sustainability, quantitative data concerning procedure, processes and costs derived from Niguarda Hospitals. On the other hand, questionnaires were administered through interviews, to healthcare professionals using the investigated technologies and involved in the UF management, referring to both Niguarda and L’Aquila Hospitals, since they were the hospitals that have gained experience in the use of MRgFUS for UF patients, in 2018.

Before starting the assessment, the PICO approach for the literature validation, in terms of “Patients”, “Intervention”, “Comparator” and “Outcomes”, was identified and discussed before setting the specific search strategy, for the HTA report. Literature evidence came from the systematic search of literature databases (Pubmed, Embase and Cochrane Library databases) up to December 2019. Search terms were: “magnetic resonance-guided (high intensity) focused ultrasound”, “MRgFUS”, “MRgHIFU”, “uterine fibroids”, “UAE”, “myomectomy”, “symptoms relief”. Papers with the following characteristics, were collected and considered: manuscripts including adult women (> 18), describing uterine fibroids symptoms, treated with magnetic resonance-guided (high intensity) focused ultrasound or with surgical approach (laparoscopy or laparotomy), and uterine fibroid embolization, in the same study. The level of evidence of the studies included in the analysis was evaluated, according to the Oxford Centre for Evidence-Based Medicine Table. Papers meeting the abovementioned inclusion criteria were consequently included and synthetized according to a PRISMA flow diagram [18], thus mapping out the number of records (in terms of papers) identified, included and/or excluded, and the reasons for exclusion. Furthermore, the assessment of the scientific evidence included in the HTA, was performed through the New Castle Ottawa Evaluation scale for cohort and observational studies, and the Cochrane risk-of-bias tool for randomized trials (RoB 2), to define the potential risk of bias [19, 20].

Literature was then used, to retrieve evidence-based information regarding the safety and the efficacy profiles of the different methodologies used in the clinical practice for the conservative treatment of patients with UFs.

For the deployment of the economic, organizational, and social dimensions, real-life data derived from the anonymous administrative and accounting flows, by cost center provided by the management control of Niguarda Hospital (Milan, Italy), referring to the UF fibroids’ management, collected from January to December 2016. The anonymous data collection was verified, approved, and validated by the Healthcare Directorate of Niguarda Hospital.

According to the above, 224 patients’ clinical pathways, derived from the observation of 224 administrative records (73 surgery; 63 UAE and 88 MRgFUS), were analyzed and economically valorized in an anonymous and aggregated manner. All the UF procedures referring to adult women undergone to surgical or interventional treatment for the removal of a maximum of three uterine fibroids, smaller than 10 cm, were considered and were economically valorized.

At first, through the implementation of an Activity Based Costing (ABC) approach [21], all the costs related to each technology were examined, considering both the surgical/interventional treatment and all the pre-operative/post-operative costs. The following items of healthcare expenditure related to each clinical pathway were considered, assuming the Italian NHS point of view: i) surgical/interventional treatment, ii) laboratory exams, iii) diagnostic and specialist procedures performed during the follow-up period after surgical or not invasive interventions, and iv) the length of stay. Only direct costs were accordingly investigated, and the total cost for each patient was calculated by multiplying the quantity of resources consumed by their unit cost. The above information was evaluated in accordance with outpatients/hospital admission reimbursement tariffs, updated to the years 2018 and valid nowadays.

The economic evaluation of the process was integrated with a budget impact analysis [22] assuming the Italian NHS perspective, to define the economic sustainability regarding the introduction of MRgFUS within the Italian clinical practice, predicting the economic and financial consequences of adopting a new technology within a healthcare organization, with finite resources. To design the budget impact analysis, a baseline scenario (or base-case scenario – Scenario 1), in which all the national patients were treated with the surgical approach, was compared to different innovative scenarios: a) the “real-life scenario” (Scenario 2), that considered the implementation of all the technologies under assessment, according to real-life practice case-mix, retrieved within the hospital (MRgFUS: 15.12%; UAE: 9.88%; Surgical setting: 75.00%); b) the “innovative scenario DH” (Scenario 3), that considered all the MRgFUS to be conducted using a day-hospital approach, on the basis of the previously mentioned case-mix of procedures.

Besides the healthcare evolution up to 12-months after surgery, the impact related to a second surgical/interventional procedure was examined, considering a different failure-rate for each technology, derived from the observation of the 224 administrative records used for the assessment of the economic dimension. A failure-rate equal to 9.78, 7.61 and 11.22%, for surgery, UAE and MRgFUS respectively, was considered.

Real-world data information, derived from the above administrative databases, were also used for the deployment of both the organizational and the social domains. On the one hand, the organizational impact was detected, in terms of organizational advantages related to the release of the operating room (OR) occupancy hours, since the innovative technology does not need to be implemented within that setting, as happened for the reference comparators (UAE and surgery). On the other hand, the social impact was assessed through the economic quantification of the patients’ productivity losses to solve their health needs, according to the different length of stay related to the three procedures under assessment.

In conclusion, a specific questionnaire was administered through structured interviews for the assessment of the ethical (in terms of accessibility to care), social and legal domains. Thus, the questionnaire was filled in by eleven experts in the treatment of uterine fibroids, who gave their comparative perceptions to the three technologies under assessment, according to an evaluation scale ranging from − 3 (less performant technology) to + 3 (most performant technology) [23]. This was useful because for under discovered research areas, the collection of healthcare professionals’ perceptions attempt to fill in gaps that are left unexposed by survey-based research, as well as literature evidence [24–26]. On the one hand, the ethical aspects explored the following items: i) Access to care on local level; ii) Access to care for person of a legally protected status; iii) Impact on the hospital waiting list; iv) Generation of health migrations; v) Existence of factors influencing the patient’s ability and autonomy; vi) Existence of factor limiting the use of the technology for a group of patients; vii) Protection of persons of a legally protected status; viii) Iniquity; ix) Influence on the patient’s dignity; x) Influence on the patient’s religion.

On the other hand, the social dimension required the professionals’ perceptions with regard to i) Ability of the technology to protect the patients’ autonomy; ii) Protection of human rights; iii) The use of technology guarantees the social values and the willingness to pay of the patient; iv) Protection of persons of a legally protected status; v) Ability of the technology to protect the patients’ religion; vi) Impact of the procedure on the social costs; vii) Patients and citizens can have a good level of understanding of technology; viii) Impact of the procedure on the patient’s perceived quality of life; ix) Impact of the procedure on the care giver’s life and perception; and x) Recovery rate.

In conclusion, the legal domain explored the following items: i) Permission level of technology; ii) Need for inclusion of the technology in registry; iii) Fulfillment of the safety requirements; iv) Infringement of intellectual property rights; v) The need to regulate the acquisition of technology; vi) The legislation covers the regulation of technology for all categories of patients; vii) The user manual of the technology is complete.

Focusing on statistical methods, data were first analyzed, considering descriptive statistics. Differences among technologies (MRgFUS, UAE and surgical setting) were evaluated, according to a significance level lower than 0.05 (*p*-value), thus using the one-way ANOVA. All the analyses were performed using the Statistical Package for Social Science of IBM SPSS (Version 22).

## Results

### Results from literature review

Focusing on the literature review, out of 236 records of studies screened, in accordance with the above proposed search strategy, only 5 [3, 27–30] met the inclusion criteria.

**Fig. 1:**
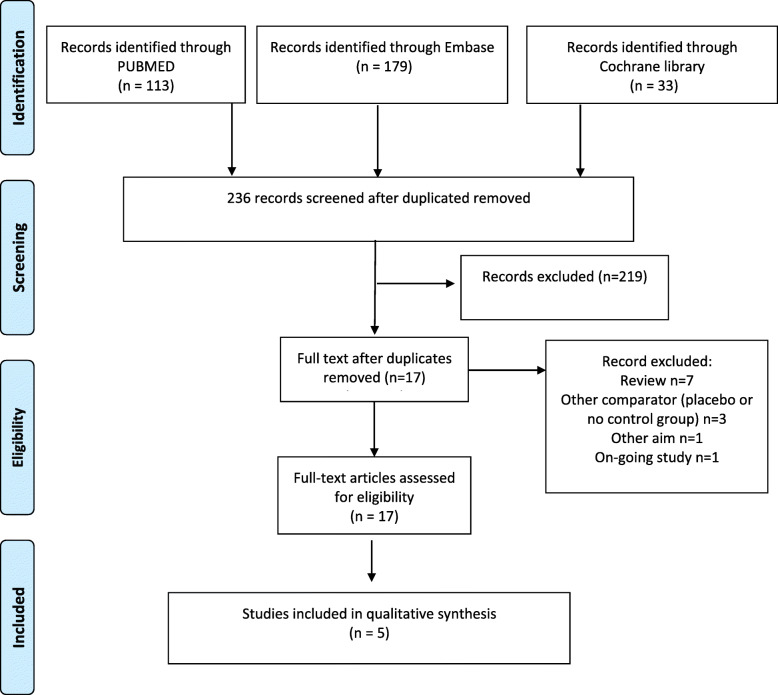
Prisma Flow Chart

The other studies were removed for the following reasons (Fig. 1): i) studies compared MRgFUS with other technologies such as placebo; ii) evidence had other aims, without focusing on efficacy/safety information; or iii) represented ongoing studies.

The implementation of both the Cochrane risk-of-bias tool for randomized trials and the Newcastle-Ottawa Scale, declared that none of the studies were at critical risk, due to selection bias, missing data and results. Thus, the risk of bias was not high. The control group was determined and the outcomes measurement proved to be relevant and replicable.

As previously mentioned, literature evidence was accordingly utilized to define the safety and the efficacy profiles of the three technologies under assessment.

Focusing on the safety domain, from an evidence-based point of view, the 92.5% of patients treated with UAE presented abdominal pain and bloating, fever, and vomiting, whereas patients treated with MRgFUS presented less post-treatment symptoms [3, 27]. Focusing on the occurrence of major complications, MRgFUS is associated with the development of fewer adverse events, reporting a significant difference if compared with the other procedures (MRgFUS: 1.3% vs UAE: 3.4% vs Surgery: 2.1%, *p* < 0.001); fewer patients in MRgFUS arm experienced infections, hemorrhages requiring infusion, unintended major surgeries, and life-threatening events [13, 31].

Focusing on the efficacy profile, the “symptoms relief” was considered the primary outcome of all the UF treatment procedures and was assessed by means of the UFS-QOL, that is a validated scale to rate disease-specific symptoms and health-related quality of life questionnaire for UF [32]. Literature evidence [10, 13, 31–34] revealed that the innovative technology would be capable to better manage the UF symptoms (MRgFUS: 0.894 vs UAE: 0.853 vs Surgery: 0.799).

### Results from the economic evaluation

The average costs related to the different clinical pathways have been estimated, considering the pre-operative and post-operative costs (also in terms of follow-up procedures). In this view, the surgical procedure absorbed more economic resources, than both MRgFUS (€ 3311.14 vs € 2937.00, *p*-value = 0.024) and UAE (€ 3311.14 vs € 2826.46 *p*-value < 0.001).

After having defined the costs related to each treatment procedure, a BIA was conducted, taking into consideration the potential Italian population eligible to UF treatments. Thus, the number of women suffering from UFs was defined, based on the disease prevalence rate, equal to 13.80%, presented in the literature evidence [5]. Considering the number of women within the Italian setting (*N* = 14,467,353 women, updated to December 2019), 1,996,495 patients emerged to be potentially treated for UFs, in the national context.

According to the above, the Italian NHS would benefit of economic advantages from the adoption of MRgFUS, ranging from 6.42 to 7.04% (Table 1), for the treatment of 1,996,495 women. The real amount of economic savings depends on the proportion of population treated with the innovation, and whether the women treated are taken in charge from the hospital, with or without hospitalization.

**Table 1:** Results from the budget impact analysis assuming the NHS point of view

	**Baseline scenario**^**1**^	**Real-life scenario**^**2**^	**Difference €**	**Difference %**
12-month clinical pathway, considering surgical procedure and follow-up	€ 5,981,802,665.58	€ 5,601,246,799.34	-€ 380,555,866.23	−6.36%
Re-intervention rate	€ 585,020,300.69	€ 544,232,524.43	-€ 40,787,776.27	−6.97%
**Total**	**€ 6,566,822,966.27**	**€ 6,145,479,323.77**	**-€ 421,343,642.50**	**−6.42%**
	**Baseline scenario**^**1**^	**Innovative scenario DH**^**3**^	**Difference €**	**Difference %**
12-month clinical pathway, considering surgical procedure and follow-up	€ 5,981,802,665.58	€ 5,563,238,083.08	-€ 418,564,582.50	−7.00%
Re-intervention rate	€ 585,020,300.69	€ 541,165,322.12	-€ 43,854,978.58	−7.50%
**Total**	**€ 6,566,822,966.27**	**€ 6,104,403,405.19**	**-€ 462,419,561.08**	**−7.04%**

^1^ The baseline scenario assumed that all the patients were treated with the surgical approach

^2^ The real-life scenario assumed the implementation of all the technologies under assessment according to real-life practice case-mix (MRgFUS: 15.12%; UAE: 9.88%; Surgical setting: 75.00%)

^3^ The real-life scenario assumed the implementation of all the technologies under assessment according to real-life practice case-mix (MRgFUS: 15.12%; UAE: 9.88%; Surgical setting: 75.00%), considering all the MRgFUS to be conducted using a day-hospital approach

It is important to notice that this saving could be reinvested for the implementation of additional DRGs related to the treatment of uterine fibroids. Considering the economic saving equal to €421,343,642.50, Italian NHS would be able to perform 131.852 additional DRGs, for UFs treatment and cure.

### Results from the organizational and social assessments

From a quantitative point of view, MRgFUS would also generate significant advantages related to the release of the operating room (OR) occupancy hours, since the innovative technology does not need to be implemented within that setting, as happened for the reference comparators (UAE and surgery), even if the innovative technology takes more than 3 h. Based on the number of patients potentially treated for uterine fibroids and evaluating the duration of a single intervention equal to 110 min for the surgical procedure and to 98 min for UAE, the OR time saving was evaluated by comparing the previously mentioned baseline scenario and the real-life scenario, achieving an organizational OR saving, equal to − 16.00% of time (Table 2).

**Table 2:** The quantitative assessment of the Organizational dimension

	Baseline scenario [minutes]	Real-life scenario [minutes]	Difference [minutes]	Difference %
**First Surgical/interventional procedure**	219,614,418.54	184,048,954.57	−35,565,463.97	−16.19%
**Re-intervention**	21,478,290.13	18,465,951.91	−3,012,338.23	−14.03%
**Total**	**241,092,708.67**	**202,514,906.47**	**−38,577,802.20**	**−16.00%**

In addition, from the analysis of the administrative databases related to the patient’s length of stay, it emerged that MRgFUS required on average 1.16 days spent in hospital after the procedure, compared with 4.03 days for surgery (*p*-value < 0.001) and 1.28 days for UAE (*p*-value > 0.05).

The implementation of MRgFUS could bring significant advantages, concerning the social aspects. On the one hand, literature [3, 27] reported that patients returned to normal life within 25 days, 10 days, and 3 days, respectively for surgical approach, UAE and MRgFUS. On the other hand, a reduction in the social costs, in terms of out-of-pocket expenditure emerged. In fact, the costs afforded by patients undergoing a surgical/interventional procedure were investigated, based on their hospital stay, thus considering both travel costs (with the inclusion of fuel and transports’ amortization costs), from home to the hospital, and the number of days spent. As a result, it emerged that the introduction of MRgFUS significantly decreases the productivity loss of a patient, reporting an average saving equal to 54.60% (€ 352.65 vs € 776.70, *p*-value < 0.001), when compared with surgery, and a saving of 20.37% (€ 352.65 vs € 442.86, *p*-value = 0.024), when compared with UAE.

### Results from the healthcare professionals’ perceptions

Table 3 depicts the results achieved from the collection of healthcare professionals’ perceptions on the equity, social, and legal dimensions, carried out by structured interviews.

**Table 3:** Perceptions on equity, social, and legal dimensions

**Equity aspects**	**MRgFUS**	**Surgery**	**UAE**	***p*-value**
Access to care on local level	−2.00	0.83	−0.83	0.022
Access to care for person of a legally protected status	−0.67	1.00	0.00	0.034
Impact on the hospital waiting list	0.17	−1.83	0.17	0.048
Generation of health migrations	2.83	0.17	1.83	0.031
Existence of factors influencing the patient’s ability and autonomy	0.83	0.00	0.67	0.286
Existence of factors limiting the use of the technology for a group of patients	0.00	0.17	0.00	0.564
Protection of persons of a legally protected status	0.17	0.00	0.00	0.331
Iniquity	0.17	0.17	0.33	0.445
Influence on the patient’s dignity	0.17	0.17	−0.17	0.321
Influence on the patient’s religion	0.00	0.00	0.00	0.786
***Average Value***	***0.17***	***0.07***	***0.20***	***0.132***
**Social aspects**	**MRgFUS**	**Surgery**	**UAE**	***p*-value**
Ability of the technology to protect the patients’ autonomy	2.50	0.33	1.50	**0.034**
Protection of human rights	0.17	0.17	0.17	**0.878**
The use of technology guarantees the social values and the willingness to pay of the patient	0.17	0.17	0.17	**0.995**
Protection of persons of a legally protected status	0.17	0.17	0.17	**0.932**
Ability of the technology to protect the patients’ religion	0.17	0.00	0.00	**0.675**
Impact of the procedure on the social costs	2.17	−0.17	1.50	**0.045**
Patients and citizens can have a good level of understanding of technology	1.17	1.33	1.17	**0.743**
Impact of the procedure on the patient’s perceived quality of life	2.67	1.00	1.67	**0.037**
Impact of the procedure on the care giver’s life and perception.	2.33	1.17	1.50	**0.028**
Recovery rate	3.00	0.33	1.83	**0.007**
***Average Value***	***1.45***	***0.45***	***0.97***	***0.044***
**Legal aspects**	**MRgFUS**	**Surgery**	**UAE**	***p*-value**
Permission level of technology	0.00	0.00	0.00	0,887
Need for inclusion of the technology in registry	0.00	0.00	0.00	0.864
Fulfillment of the safety requirements	0.67	0.67	0.67	0.941
Infringement of intellectual property rights	0.00	0.00	0.00	0.898
The need to regulate the acquisition of technology	−0.17	−0.17	−0.17	0.886
The legislation covers the regulation of technology for all categories of patients	0.00	0.00	0.00	0.911
The user manual of the technology is complete	0.83	0.17	0.83	0.314
***Average Value***	***0.19***	***0.10***	***0.19***	***0.648***

From an equity perspective, it emerged that UAE achieved the better perception, and should be considered the preferable technology, followed by MRgFUS, albeit not statistically differences occurred between alternatives (*p*-value > 0.05). MRgFUS is not always accessible on local level, since not all the hospitals have, to date, acquired the innovative large size equipment useful for the implementation of guided-ultrasound procedures. However, the adoption of MRgFUS could generate health migration phenomena (*p*-value = 0.031). In particular, the choice of providing a larger number of technological alternatives is relevant for the increase in the number of procedures and extension of the catchment area of the reference, thus indicating how the innovative technology could increase, in the future, healthcare migration and mobility.

As for the quantitative analysis of the social dimension, professionals agreed that MRgFUS would be the preferable solution (*p*-value = 0.044), regarding an increase in the quality of life perceived by the patients themselves (*p*-value = 0.037), and by their families (*p*-value = 0.028), as well as a faster return to regular working activities (*p*-value = 0.007).

The legal impact examination reported no statistically significant differences among technologies considering the indication of use, for all the surgical/interventional procedures, and for all the categories of patients (MRgFUS = 0.19 vs UAE = 0.19 vs Surgery = 0.10, *p*-value > 0.05). In addition, concerning the two minimally invasive techniques, the professionals involved, declared the completeness and integrity of the user manuals.

## Discussion

The results of the study show that MRgFUS may be considered a valid technological alternative within the specific setting of uterine fibroids’ treatment, to be offered to women who meet the inclusion criteria, providing a potential overall benefit with its acquisition. Relevant MRgFUS strengths are found in almost every investigated dimension. It should be noted that UAE could also be considered as another mini-invasive valid treatment option.

From an efficacy and safety perspective, there emerged the good profile of minimally invasive technologies, in terms of better symptoms control, as well as lower occurrence rate of severe adverse events, with important benefits also in terms of quality of life [7, 35]. These advantages may result in economic benefits from both a payer’s and a societal perspective. Thus, the introduction of such alternative technologies would give an advantage in all the scenarios analyzed, when compared with the baseline situation consisting of only use of surgery for the removal of uterine fibroids. In particular, the greater the number of patients being treated with MRgFUS, the higher the economic advantage registered. Assuming the patients’ point of view, both UAE and MRgFUS could be considered as the preferable treatment option, particularly in terms of reduced length of stay, with an important saving in the overall patients’ productivity losses due to their clinical condition. In fact, patients would have a higher perception on their quality of life and would return faster to normal life and/or work, with a consequent decrease in productivity loss. In this view, literature reported that MRgFUS is associated with cost savings and a small QALY improvement, if compared with the current practice [11, 12, 36, 37]: this confirms not only its effectiveness, but also the possibility to have good economic results as well. The economic results achieved, in terms of solely economic evaluation of the process, was consistent with literature evidence available on the topic, that report a mean cost per patient was €3249.31, with a median of €2978.71, but considering in general surgical (any type) and non-surgical patients [31].

Moving on from these premises, the analysis of the results would lead to the MRgFUS introduction as an outpatient procedure, as revealed in other settings such as United States [13]. Thus, the analysis, albeit conducted using real-world data and perception of a specific Italian setting, may have important implications for decision-makers, since disinvestment and costs reduction are always priorities on the policy and decision makers agendas, particularly in the current era of economic recession. Strategies are needed to reduce costs, preserving effectiveness and appropriateness, and guaranteeing universality and equity of care in the National Healthcare Services. In this view, MRgFUS adoption could be associated with large cost-savings when introduced into healthcare systems, both from an economic and from an organizational point of view, with the ability also to improve the system capacity and hospital waiting lists, a growing problem for Italian and European hospitals.

Anyway, it should be noted that further information is needed to enrich the results. The failure rate of intervention, with the consequent necessity for the patient to undergo to a second interventional procedure, was collected within one hospital performing MRgFUS, with a strong impact on both economic and social dimension in terms of costs, influencing the whole technology assessment. Assuming a 12-month observation, the failure rate related to MRgFUS use was equal to 11.22%, and a similar rate is registered in other evidence, with a value of 12.7 and 11% [38, 39]. Other studies reported a quite different failure rate, such as 4% [30] or 24% [39], even if they considered a different follow-up period (6 months and 5 years, respectively). Furthermore, to date there are no randomized clinical trials, which would provide more robust information.

Despite further real-life evidence should be collected and further controlled and randomized studies will be required, MRgFUS is an effective and promising therapeutic technique for decreasing myoma volume and patients’ symptoms.

## Conclusions

Uterine fibroids are the direct cause of a significant healthcare burden for women, their families and society. In the clinical practice, surgical myomectomy remains the gold standard for treating reproductive-age women. However, in the recent years, the wide evolution of less invasive approaches led to a change in the options used by the clinician to treat symptomatic fibroids. Minimally invasive procedures such as uterine artery embolization (UAE) are increasingly used to treat symptomatic fibroids. Indeed, the selection of the proper methods to be preceded by a thorough analysis of the case, patient’s age, tumor location and related symptoms.

In this view, MRgFUS may constitute an alternative solution for patients who meet the qualification criteria and deny other methods, which also facilitates the use of other treatment options in case the procedure is ineffective, with important organizational, economic, and social benefits, even if further randomized studies are necessary, to confirm the above information.

In conclusions, the healthcare services may consider the evidence provided by the present study as an opportunity to differentiate UFs patients’ procedures, guaranteeing a personalized clinical pathway and different alternatives, thus becoming more efficient and effective.

## Supplementary Information

**Additional file 1.**

**Additional file 2.**

## Data Availability

The datasets used and/or analysed during the current study are available from the corresponding author on reasonable request.

## Abbreviations

BIA: Budget Impact Analysis
HTA: Health Technology Assessment
MRgFUS: Magnetic Resonance-guided Focused Ultrasound
NHS: National Health Service
QALY: Quality Adjusted Life Years
UAE: Uterine artery embolization
UF: Uterine Fibroids
