# Supplementary material for: Innovative and conventional “conservative” technologies for the treatment of uterine fibroids in Italy: a multidimensional assessment
Source: Health Econ Rev. 2022 Mar 18;12:21. doi: 10.1186/s13561-022-00367-x (PMC8932203; doi:10.1186/s13561-022-00367-x)
Supplement: Supplementary file 1 — Additional file 1. [file 13561_2022_367_MOESM1_ESM.docx]

**QUESTIONNAIRE**

**Equity impact**

Dear Doctor,

the questionnaire that we ask you to fill in, aims at evaluating the perceived equity impact (in terms of accessibility to care for patients suffering from uterine fibroids) related to the use of the different conservative procedures, available nowadays in the clinical practice, and considering the potential introduction of the innovative Magnetic Resonance-guided high intensity Focused Ultrasound.

On the basis of your own experience and perceptions, we kindly ask you to evaluate every single item, in accordance to a 7-item Likert scale, ranging from -3 to + 3 (impact index: 0: null; +1: low positive; + 2: medium positive; +3: high positive; -1 negative low; -2: negative average; -3: high negative).)

We ask you to indicate the answer that seems most appropriate to you.

Data will be investigated using aggregated methods. Information will be confidential, in accordance with the EU Regulation n. 679 of 04.05.2016.

Thank you in advance for your support.

***Access to care on local level***

| *MRgFUS* | -3 | -2 | -1 | 0 | 1 | 2 | 3 |
| --- | --- | --- | --- | --- | --- | --- | --- |
| *Uterine Artery Embolisation (UAE)* | -3 | -2 | -1 | 0 | 1 | 2 | 3 |
| *Myomectomy (standard procedure)* | -3 | -2 | -1 | 0 | 1 | 2 | 3 |

***Access to care for person of a legally protected status***

| *MRgFUS* | -3 | -2 | -1 | 0 | 1 | 2 | 3 |
| --- | --- | --- | --- | --- | --- | --- | --- |
| *Uterine Artery Embolisation (UAE)* | -3 | -2 | -1 | 0 | 1 | 2 | 3 |
| *Myomectomy (standard procedure)* | -3 | -2 | -1 | 0 | 1 | 2 | 3 |

***Impact on the hospital waiting list***

| *MRgFUS* | -3 | -2 | -1 | 0 | 1 | 2 | 3 |
| --- | --- | --- | --- | --- | --- | --- | --- |
| *Uterine Artery Embolisation (UAE)* | -3 | -2 | -1 | 0 | 1 | 2 | 3 |
| *Myomectomy (standard procedure)* | -3 | -2 | -1 | 0 | 1 | 2 | 3 |

***Generation of health migrations***

| *MRgFUS* | -3 | -2 | -1 | 0 | 1 | 2 | 3 |
| --- | --- | --- | --- | --- | --- | --- | --- |
| *Uterine Artery Embolisation (UAE)* | -3 | -2 | -1 | 0 | 1 | 2 | 3 |
| *Myomectomy (standard procedure)* | -3 | -2 | -1 | 0 | 1 | 2 | 3 |

***Existence of factors influencing the patient’s ability and autonomy***

| *MRgFUS* | -3 | -2 | -1 | 0 | 1 | 2 | 3 |
| --- | --- | --- | --- | --- | --- | --- | --- |
| *Uterine Artery Embolisation (UAE)* | -3 | -2 | -1 | 0 | 1 | 2 | 3 |
| *Myomectomy (standard procedure)* | -3 | -2 | -1 | 0 | 1 | 2 | 3 |

***Existence of factor limiting the use of the technology for a group of patients***

| *MRgFUS* | -3 | -2 | -1 | 0 | 1 | 2 | 3 |
| --- | --- | --- | --- | --- | --- | --- | --- |
| *Uterine Artery Embolisation (UAE)* | -3 | -2 | -1 | 0 | 1 | 2 | 3 |
| *Myomectomy (standard procedure)* | -3 | -2 | -1 | 0 | 1 | 2 | 3 |

***Protection of persons of a legally protected status***

| *MRgFUS* | -3 | -2 | -1 | 0 | 1 | 2 | 3 |
| --- | --- | --- | --- | --- | --- | --- | --- |
| *Uterine Artery Embolisation (UAE)* | -3 | -2 | -1 | 0 | 1 | 2 | 3 |
| *Myomectomy (standard procedure)* | -3 | -2 | -1 | 0 | 1 | 2 | 3 |

***Iniquity***

| *MRgFUS* | -3 | -2 | -1 | 0 | 1 | 2 | 3 |
| --- | --- | --- | --- | --- | --- | --- | --- |
| *Uterine Artery Embolisation (UAE)* | -3 | -2 | -1 | 0 | 1 | 2 | 3 |
| *Myomectomy (standard procedure)* | -3 | -2 | -1 | 0 | 1 | 2 | 3 |

***Influence on the patient’s dignity***

| *MRgFUS* | -3 | -2 | -1 | 0 | 1 | 2 | 3 |
| --- | --- | --- | --- | --- | --- | --- | --- |
| *Uterine Artery Embolisation (UAE)* | -3 | -2 | -1 | 0 | 1 | 2 | 3 |
| *Myomectomy (standard procedure)* | -3 | -2 | -1 | 0 | 1 | 2 | 3 |

***Influence on the patient’s religion***

| *MRgFUS* | -3 | -2 | -1 | 0 | 1 | 2 | 3 |
| --- | --- | --- | --- | --- | --- | --- | --- |
| *Uterine Artery Embolisation (UAE)* | -3 | -2 | -1 | 0 | 1 | 2 | 3 |
| *Myomectomy (standard procedure)* | -3 | -2 | -1 | 0 | 1 | 2 | 3 |

**Social impact**

Dear Doctor,

the questionnaire that we ask you to fill in, aims at evaluating the perceived social impact (assuming the patients’ perspective) related to the use of the different conservative procedures, available nowadays in the clinical practice, and considering the potential introduction of the innovative Magnetic Resonance-guided high intensity Focused Ultrasound.

On the basis of your own experience and perceptions, we kindly ask you to evaluate every single item, in accordance to a 7-item Likert scale, ranging from -3 to + 3 (impact index: 0: null; +1: low positive; + 2: medium positive; +3: high positive; -1 negative low; -2: negative average; -3: high negative).)

We ask you to indicate the answer that seems most appropriate to you.

Data will be investigated using aggregated methods. Information will be confidential, in accordance with the EU Regulation n. 679 of 04.05.2016.

Thank you in advance for your support.

***Ability of the technology to protect the patients’ autonomy***

| *MRgFUS* | -3 | -2 | -1 | 0 | 1 | 2 | 3 |
| --- | --- | --- | --- | --- | --- | --- | --- |
| *Uterine Artery Embolisation (UAE)* | -3 | -2 | -1 | 0 | 1 | 2 | 3 |
| *Myomectomy (standard procedure)* | -3 | -2 | -1 | 0 | 1 | 2 | 3 |

***Protection of human rights***

| *MRgFUS* | -3 | -2 | -1 | 0 | 1 | 2 | 3 |
| --- | --- | --- | --- | --- | --- | --- | --- |
| *Uterine Artery Embolisation (UAE)* | -3 | -2 | -1 | 0 | 1 | 2 | 3 |
| *Myomectomy (standard procedure)* | -3 | -2 | -1 | 0 | 1 | 2 | 3 |

***The use of technology guarantees the social values and the willingness to pay of the patient***

| *MRgFUS* | -3 | -2 | -1 | 0 | 1 | 2 | 3 |
| --- | --- | --- | --- | --- | --- | --- | --- |
| *Uterine Artery Embolisation (UAE)* | -3 | -2 | -1 | 0 | 1 | 2 | 3 |
| *Myomectomy (standard procedure)* | -3 | -2 | -1 | 0 | 1 | 2 | 3 |

***Protection of persons of a legally protected status***

| *MRgFUS* | -3 | -2 | -1 | 0 | 1 | 2 | 3 |
| --- | --- | --- | --- | --- | --- | --- | --- |
| *Uterine Artery Embolisation (UAE)* | -3 | -2 | -1 | 0 | 1 | 2 | 3 |
| *Myomectomy (standard procedure)* | -3 | -2 | -1 | 0 | 1 | 2 | 3 |

***Ability of the technology to protect the patients’ religion***

| *MRgFUS* | -3 | -2 | -1 | 0 | 1 | 2 | 3 |
| --- | --- | --- | --- | --- | --- | --- | --- |
| *Uterine Artery Embolisation (UAE)* | -3 | -2 | -1 | 0 | 1 | 2 | 3 |
| *Myomectomy (standard procedure)* | -3 | -2 | -1 | 0 | 1 | 2 | 3 |

***Impact of the procedure on the social costs***

| *MRgFUS* | -3 | -2 | -1 | 0 | 1 | 2 | 3 |
| --- | --- | --- | --- | --- | --- | --- | --- |
| *Uterine Artery Embolisation (UAE)* | -3 | -2 | -1 | 0 | 1 | 2 | 3 |
| *Myomectomy (standard procedure)* | -3 | -2 | -1 | 0 | 1 | 2 | 3 |

***Patients and citizens can have a good level of understanding of technology***

| *MRgFUS* | -3 | -2 | -1 | 0 | 1 | 2 | 3 |
| --- | --- | --- | --- | --- | --- | --- | --- |
| *Uterine Artery Embolisation (UAE)* | -3 | -2 | -1 | 0 | 1 | 2 | 3 |
| *Myomectomy (standard procedure)* | -3 | -2 | -1 | 0 | 1 | 2 | 3 |

***Impact of the procedure on the patient’s perceived quality of life***

| *MRgFUS* | -3 | -2 | -1 | 0 | 1 | 2 | 3 |
| --- | --- | --- | --- | --- | --- | --- | --- |
| *Uterine Artery Embolisation (UAE)* | -3 | -2 | -1 | 0 | 1 | 2 | 3 |
| *Myomectomy (standard procedure)* | -3 | -2 | -1 | 0 | 1 | 2 | 3 |

***Impact of the procedure on the care giver’s life and perception***

| *MRgFUS* | -3 | -2 | -1 | 0 | 1 | 2 | 3 |
| --- | --- | --- | --- | --- | --- | --- | --- |
| *Uterine Artery Embolisation (UAE)* | -3 | -2 | -1 | 0 | 1 | 2 | 3 |
| *Myomectomy (standard procedure)* | -3 | -2 | -1 | 0 | 1 | 2 | 3 |

***Recovery rate***

| *MRgFUS* | -3 | -2 | -1 | 0 | 1 | 2 | 3 |
| --- | --- | --- | --- | --- | --- | --- | --- |
| *Uterine Artery Embolisation (UAE)* | -3 | -2 | -1 | 0 | 1 | 2 | 3 |
| *Myomectomy (standard procedure)* | -3 | -2 | -1 | 0 | 1 | 2 | 3 |

**Legal impact**

Dear Doctor,

the questionnaire that we ask you to fill in, aims at evaluating the perceived legal impact, related to the use of the different conservative procedures, available nowadays in the clinical practice, and considering the potential introduction of the innovative Magnetic Resonance-guided high intensity Focused Ultrasound.

On the basis of your own experience and perceptions, we kindly ask you to evaluate every single item, in accordance to a 7-item Likert scale, ranging from -3 to + 3 (impact index: 0: null; +1: low positive; + 2: medium positive; +3: high positive; -1 negative low; -2: negative average; -3: high negative).)

We ask you to indicate the answer that seems most appropriate to you.

Data will be investigated using aggregated methods. Information will be confidential, in accordance with the EU Regulation n. 679 of 04.05.2016.

Thank you in advance for your support.

***Permission level of technology***

| *MRgFUS* | -3 | -2 | -1 | 0 | 1 | 2 | 3 |
| --- | --- | --- | --- | --- | --- | --- | --- |
| *Uterine Artery Embolisation (UAE)* | -3 | -2 | -1 | 0 | 1 | 2 | 3 |
| *Myomectomy (standard procedure)* | -3 | -2 | -1 | 0 | 1 | 2 | 3 |

***Need for inclusion of the technology in registry***

| *MRgFUS* | -3 | -2 | -1 | 0 | 1 | 2 | 3 |
| --- | --- | --- | --- | --- | --- | --- | --- |
| *Uterine Artery Embolisation (UAE)* | -3 | -2 | -1 | 0 | 1 | 2 | 3 |
| *Myomectomy (standard procedure)* | -3 | -2 | -1 | 0 | 1 | 2 | 3 |

***Fulfillment of the safety requirements***

| *MRgFUS* | -3 | -2 | -1 | 0 | 1 | 2 | 3 |
| --- | --- | --- | --- | --- | --- | --- | --- |
| *Uterine Artery Embolisation (UAE)* | -3 | -2 | -1 | 0 | 1 | 2 | 3 |
| *Myomectomy (standard procedure)* | -3 | -2 | -1 | 0 | 1 | 2 | 3 |

***Infringement of intellectual property rights***

| *MRgFUS* | -3 | -2 | -1 | 0 | 1 | 2 | 3 |
| --- | --- | --- | --- | --- | --- | --- | --- |
| *Uterine Artery Embolisation (UAE)* | -3 | -2 | -1 | 0 | 1 | 2 | 3 |
| *Myomectomy (standard procedure)* | -3 | -2 | -1 | 0 | 1 | 2 | 3 |

***The need to regulate the acquisition of technology***

| *MRgFUS* | -3 | -2 | -1 | 0 | 1 | 2 | 3 |
| --- | --- | --- | --- | --- | --- | --- | --- |
| *Uterine Artery Embolisation (UAE)* | -3 | -2 | -1 | 0 | 1 | 2 | 3 |
| *Myomectomy (standard procedure)* | -3 | -2 | -1 | 0 | 1 | 2 | 3 |

***The legislation covers the regulation of technology for all categories of patients***

| *MRgFUS* | -3 | -2 | -1 | 0 | 1 | 2 | 3 |
| --- | --- | --- | --- | --- | --- | --- | --- |
| *Uterine Artery Embolisation (UAE)* | -3 | -2 | -1 | 0 | 1 | 2 | 3 |
| *Myomectomy (standard procedure)* | -3 | -2 | -1 | 0 | 1 | 2 | 3 |

***The user manual of the technology is complete***

| *MRgFUS* | -3 | -2 | -1 | 0 | 1 | 2 | 3 |
| --- | --- | --- | --- | --- | --- | --- | --- |
| *Uterine Artery Embolisation (UAE)* | -3 | -2 | -1 | 0 | 1 | 2 | 3 |
| *Myomectomy (standard procedure)* | -3 | -2 | -1 | 0 | 1 | 2 | 3 |
