# Supplementary material for: Innovative and conventional “conservative” technologies for the treatment of uterine fibroids in Italy: a multidimensional assessment
Source: Health Econ Rev. 2022 Mar 18;12:21. doi: 10.1186/s13561-022-00367-x (PMC8932203; doi:10.1186/s13561-022-00367-x)
Supplement: Supplementary file 2 — Additional file 2. [file 13561_2022_367_MOESM2_ESM.docx]

**Table of Abstacts**

| **N** | **Authors & Study** | **Procedures performered** | **Outcome** | **Results** | **Authors’ Conclusion** | **Level of Evidence**  **&**  **Risk of bias assessment** |
| --- | --- | --- | --- | --- | --- | --- |
| 1 | Barnard, E. P., A. M. AbdElmagied, et al. 2016  **Recovery trajectory in a randomized controlled trial (RCT) of uterine artery embolization (UAE) and MR-guided focused ultrasound (FUS).** | Uterine artery embolization (UAE) and MRI guided focused  ultrasound (MRgFUS). | Safety profile, pain outcome, use of medication, days to back to normal life | 83 women underwent treatment and 75 completed the diaries.   1. No difference in incidence or severity in AEs between arms. 2. Women undergoing UAE were more likely to use outpatient pain medication, opioids or NSAID (p<0.001) and had a longer recovery. 3. the treatment arms were similar with the exception that MRgFUS women had higher pain scores at enrollment. | UAE and MRgFUS have comparable safety profiles in short term follow-up; however, women undergoing UAE had a longer recovery and required more analgesics. Baseline pain level affected outcomes for these fibroid therapies. | Level of Evidence: 1  Assessment of evidence quality by means of Cochrane risk-of-bias tool for randomized trials:  • Random sequence generation: low risk of bias  • Allocation concealment: low risk of bias  • Blinding of participants and personnel: unclear risk of bias  • Blinding of outcome assessment: unclear risk of bias  • Incomplete outcome data: low risk of bias  • Selective reporting: low risk of bias  • Other bias: low risk of bias |
| 2 | Ferrari, F., A. V. Giordano, et al. 2015  **Magnetic resonance-guided focus ultrasound surgery (MRgFUS) compared to uterine artery embolization (UAE): Main differences, advantages, therapeutic response, and definition of selection criteria** | Uterine artery embolization (UAE) and MRI guided focused  ultrasound (MRgFUS). | non-perfused volume extent, reabsorption time, clinical response, complications, and hospitalization time. | 65 patients were treated for uterine fibroids; 38 of them were treated using MRgFUS and 27 with UAE.   1. Not perfused-volume extent mean value: 95% UAE, 91.5% MRgFUS. 2. A reabsorption of the necrotic area of 50%- 70% in both techniques. 3. complications: 92.5% treated with UAE presented abdominal pain and bloating, fever, and vomiting, with a mean hospitalization time of 3 days and returned to a normal life in 25 days. Only 2 out of 27 (7.5%) returned to a normal life in 10 days. Patients treated with MRgFUS had less post-treatment symptoms, no complications, and a mean hospitalization time of 1 day and returned to a normal life in 5 days. | UAE is more radical; it seems to have a shorter reabsorption time but a longer convalescence. MRgFUS is more repeatable with a good clinical response and should be the first choice when possible. | Level of Evidence: 3  Assessment of evidence quality by means of NewCastle-Ottawa quality assessment scale:  Selection: 3/4  Comparability: ½  Outcome: 3/3 |
| 3 | Fröling, V., K. Meckelburg, et al 2012  **Comparing outcomes of magnetic resonance-guided focused ultrasound surgery and uterine artery embolization for uterine fibroids-short-term and mid-term results** | Uterine artery embolization (UAE) and MRI guided focused  ultrasound (MRgFUS) | compare the short- and mid-term improvement in Symptom Severity (SS). Health-Related Quality of Life (HRQoL) and re-intervention rate after Uterine Artery Embolization (UAE) and Magnetic Resonance-guided Focused Ultrasound Surger | 80 women MRgFUS or UAE between 2002 and 2009 and were subdivided into a short-term (n=72) and a mid-term (n=60) follow-up group.   1. Re-intervention rate after UAE in short-term follow-up was significantly lower than after MRgFUS (P< 0.001) and nearly reached significance in mid-term follow-up (P=0.063). 2. SS and HRQoL in short term follow-up after UAE, was significantly better than after MRgFUS (P=0.020; P=0.025). | UAE for symptomatic uterine fibroids has a significantly lower re-intervention rate and is superior to MRgFUS regarding symptom control and improvement in HRQL at short-term followup. At mid-term follow-up, the need for a second intervention did not differ significantly but showed a tendency to do so, while symptoms and HRQL were comparable in treatment responders. | Level of Evidence: 4  Assessment of evidence quality by means of NewCastle-Ottawa quality assessment scale:  Selection: 4/4  Comparability: 1/2  Exposure: 3/3 |
| 4 | Johnson, L. N. C., M. L. Leong, et al. 2009  **A prospective case-matched cohort analysis of magnetic resonance-guided focused ultrasound versus uterine artery embolization for treatment of symptomatic uterine fibroids**." | Uterine artery embolization (UAE) and MRI guided focused  ultrasound (MRgFUS) | patients' scores pre- and post-procedure on the Uterine Fibroid Symptom and Health-Related Quality of Life Questionnaire (UFS-QOL). need for further treatment and patient satisfaction with treatment | 15 patients who underwent MRgFUS were compared with 30 patients who underwent UAE from January 2007 to March 2009.   1. All patients in the MRgFUS group reported a ≥ 10-point decrease in the Symptom Severity Score (SSS) compared to 87% (20/23) of patients in the UAE group. After treatment, UAE patients scored significantly higher than MRgFUS patients on four out of the eight UFS-QOL subscores: Concern, Self-consciousness, Sexual function, and Total health related quality of life. Patients in the MRgFUS group had significantly larger fibroids (8.6 ±2.0 cm) than UAE patients (6.8 ± 2.5 cm, p=0.0131). | Both MRgFUS and UAE are effective, uterine-sparing fibroid treatment options that demonstrate significant symptomatic improvement in quality of life’ scores as defined by a 10-point decrease in SSS on the UFS-QOL. Further studies are needed to determine if clinical outcomes are impacted by patient selection and fibroid characteristics. In this study, women who chose to have MRgFUS had significantly larger fibroids, which may have contributed to a more modest improvement in symptom scores. | Level of Evidence: 4  Assessment of evidence quality by means of NewCastle-Ottawa quality assessment scale:  Selection: 3/4  Comparability: 1/2  Outcome: 3/3 |
| 5 | Taran, F. A., C. M. Tempany, et al. 2009  Magnetic resonance-guided focused ultrasound (MRgFUS) compared with abdominal hysterectomy for treatment of uterine leiomyomas." | MRI guided focused  ultrasound (MRgFUS) and hysterectomy | 1. Rate of adverse events 2. Rate of Significant clinical complications (SCC): as fever> 38◦ C on any 2 post-treatment days, blood transfusion, unintended major surgical procedure, discharge to a rehabilitation facility, discharge with an appliance such as a drain or urinary catheter, outpatient interventional treatment, rehospitalization, life-threatening event or death within 42 days of treatment. | A total of 192 women were included in the study, of whom 109 underwent MRgFUS and 83 underwent total abdominal hysterectomy.   1. At least one adverse event was reported by 88 (81%) women from the MRgFUS group and by 82 (99%) women from the hysterectomy group (P < 0.0001) 2. The safety profile of MRgFUS compared favorably with that of hysterectomy, with fewer SCCs occurring in the MRgFUS arm compared to the hysterectomy arm (14 events vs. 33 events; P < 0.0001). 3. At 1 month, women undergoing MRgFUS reported 1.2 lost working days and 2.7 days kept from usual activities compared with 19.2 and 17.4, respectively, for women undergoing hysterectomy (P < 0.0001). | The results of this study show that MRgFUS treatment of uterine leiomyomas leads to clinical improve- ment with fewer significant clinical complications and adverse events compared to hysterectomy at 6 months’ follow-up. | Level of Evidence: 3  Assessment of evidence quality by means of NewCastle-Ottawa quality assessment scale:  Selection: 4/4  Comparability: 2/2  Outcome: 3/3 |
